# Accurate measurement of microsatellite length by disrupting its tandem repeat structure

**DOI:** 10.1093/nar/gkac723

**Published:** 2022-09-12

**Authors:** Zihua Wang, Andrea B Moffitt, Peter Andrews, Michael Wigler, Dan Levy

**Affiliations:** Cold Spring Harbor Laboratory, Cold Spring Harbor, NY 11724, USA; Cold Spring Harbor Laboratory, Cold Spring Harbor, NY 11724, USA; Cold Spring Harbor Laboratory, Cold Spring Harbor, NY 11724, USA; Cold Spring Harbor Laboratory, Cold Spring Harbor, NY 11724, USA; Cold Spring Harbor Laboratory, Cold Spring Harbor, NY 11724, USA

## Abstract

Tandem repeats of simple sequence motifs, also known as microsatellites, are abundant in the genome. Because their repeat structure makes replication error-prone, variant microsatellite lengths are often generated during germline and other somatic expansions. As such, microsatellite length variations can serve as markers for cancer. However, accurate error-free measurement of microsatellite lengths is difficult with current methods precisely because of this high error rate during amplification. We have solved this problem by using partial mutagenesis to disrupt enough of the repeat structure of initial templates so that their sequence lengths replicate faithfully. In this work, we use bisulfite mutagenesis to convert a C to a U, later read as T. Compared to untreated templates, we achieve three orders of magnitude reduction in the error rate per round of replication. By requiring agreement from two independent first copies of an initial template, we reach error rates below one in a million. We apply this method to a thousand microsatellite loci from the human genome, revealing microsatellite length distributions not observable without mutagenesis.

## INTRODUCTION

Tumors have genomic variants that distinguish them from the host genome. These include single nucleotide variants (SNVs), small indels, large scale copy number variation (CNVs), and microsatellite length variation (MSLV). Microsatellites, tandem repeats of simple sequence units, are abundant in the genome. Their repeat structure makes them error prone during replication, resulting in variation in the number of repeat units and hence length. MSLV is very abundant in the cancers of patients with defects in mismatch repair (1,2), but also in cancers in general (3–6). Therefore, MSLV can serve as markers for cancer with value in outcome prediction, monitoring minimal residual disease, and early detection. Measuring MSLV accurately would open an efficient way to fingerprint a cancer and to detect its presence in clinical specimens (7,8). It also would impact the study of somatic phylogeny (9,10), non-malignant clonal expansions (11–13) and disease-associated microsatellites (14,15).

Unfortunately, the same property that makes microsatellites useful as markers makes them difficult to measure. The microsatellite can expand or contract by units of the repeat due to polymerase slippage during amplification also called ‘stutter’ (16–19). This is particularly problematic when measuring the lengths of mononucleotide repeats, which are the most variable type of repeat in cancers (3–6). Worse still, modern day high-throughput sequence platforms fail to read through mononucleotide tracts accurately (20,21).

Various approaches have been taken to tame this problem. Multiplex PCR and capillary electrophoresis methods have been described to measure 5–10 microsatellite loci (22–24). PCR-free (25) or isothermal amplification (26) have been demonstrated to significantly reduce stutter. MS lengths have been characterized from gene panels and high throughput sequencing (HTS) data (7,27), and statistical methods have been developed to increase accuracy in calling MS lengths from standard HTS data (25,28–31). Droplet digital PCR has been employed (8) to increase accuracy for small numbers of loci. None of these methods have the scale, depth, generality and accuracy needed for routinely monitoring a large panel of microsatellite loci deeply. Most importantly, while these other methods may suffice for genotyping homozygous and heterozygous loci, we are interested in a method that can measure and detect low frequency variants.

We meet this challenge by partial random mutagenesis of templates (32) and infer length from the reads containing the microsatellite only when the mutagenesis disrupts its repeat structure sufficiently. We use two different computational frameworks to assess the accuracy of microsatellite length measurement. We also reduce sequence error with varietal tags (33–35). In the implementation described in the Results, our experimental setup has two parts: (1) synthetic templates with well-controlled lengths and compositions, and (2) biological templates which vary significantly in length and composition. We explore the performance characteristics of the system in part 1, demonstrate applicability to real-world information in part 2, and use the similarity of behavior across parts 1 and 2 to establish the validity of extrapolation. Using synthetic templates, we analyze three different microsatellites: mononucleotide tracts containing A or C, and a dinucleotide tract with a CA repeat. For the MS containing C, we randomly deaminate a proportion of the C’s to U’s, later read as a T, with a partial bisulfite reaction (36–38). We demonstrate the presence of synthetic variants. For biological templates we use DNA from a human cell-line and enrich for 1260 microsatellite loci. We demonstrate microsatellite lengths that are unobservable without mutagenesis. We conclude with a discussion of the myriad potential applications of this method, and the obstacles that remain.

## MATERIALS AND METHODS

### Template design

**Figure 1.**
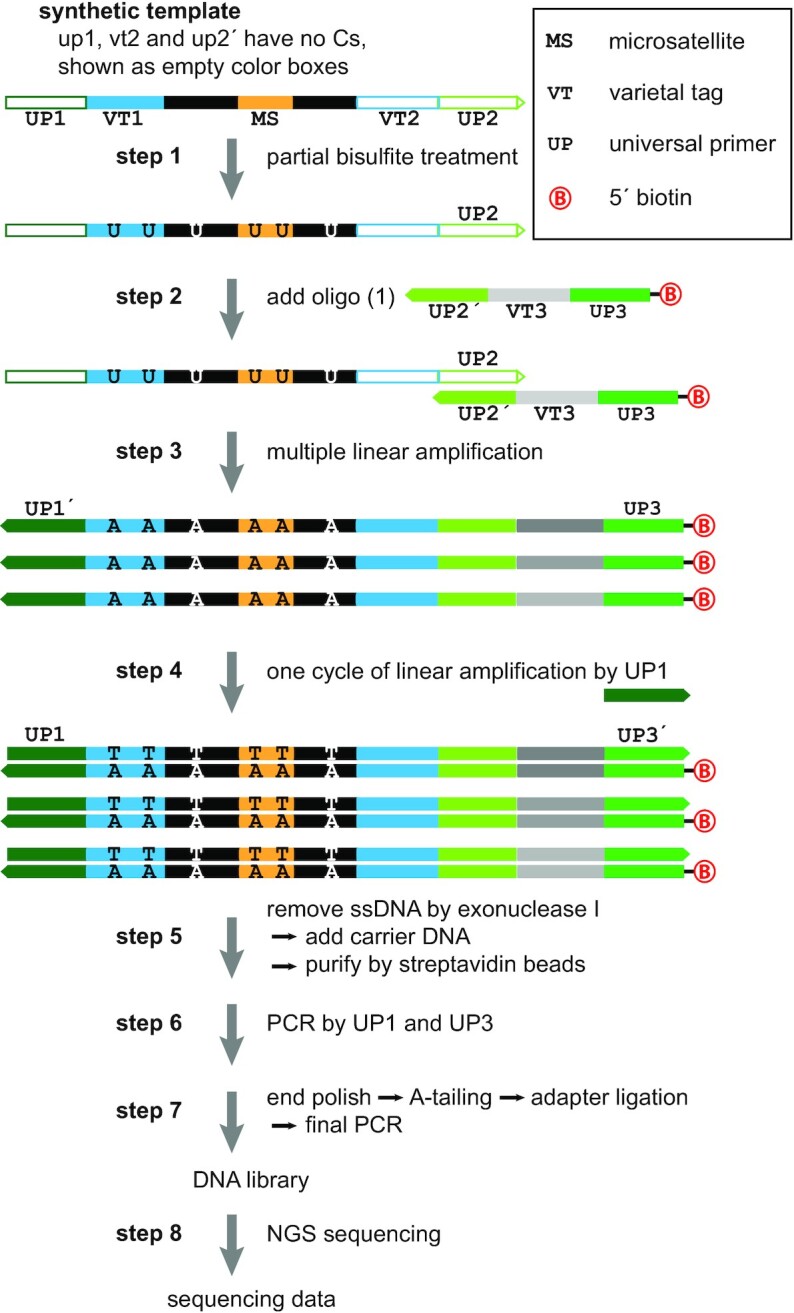
Bench protocol for partial mutagenesis. In step 1, each of the two synthetic templates containing C was partially bisulfite converted. In steps 2–3, about 6 × 10^4^ of these templates underwent 9 cycles of linear amplification by using a biotinylated oligo. In step 4, double-stranded DNA fragments were obtained in another round of linear amplification by using UP1. In step 5, extra free oligo was removed by exonuclease I. After adding carrier DNA, biotinylated DNA fragments were purified by streptavidin beads. In step 6, the exponential PCR was carried out using UP1 and UP3 to generate enough material to prepare the DNA libraries for sequencing. These were sequenced as 2 × 150 bp paired-end runs on MiSeq (steps 7–8).

For the testing and development of this method, we used three synthetic templates containing microsatellite (MS) tracts. The MS sequences are: a 17 base-pair mononucleotide A repeat called M-17 (A), an 18 base-pair mononucleotide C repeat called M-18 (C), and a 26 base-pair dinucleotide CA repeat called D-26 (CA). The templates were ordered from Integrated DNA Technologies (IDT). The full sequences of the synthetic templates, oligonucleotide adaptors, and primers are given in Supplementary Table S1. As shown in Figure 1, the structure of synthetic templates used for the partial mutagenesis protocol, M-18 (C) and D-26 (CA), is as follows: a 5′ primer binding site without cytosine (UP1), a 15-mer varietal tag sequence (VT1) with random nucleotides represented as ‘NNN…’, a 5′ flanking sequence, a C or CA microsatellite tract, another 3′ flanking sequence, another 15-mer varietal tag (VT2) with random nucleotides represented as ‘DDD…’, and finally a 3′ binding site (UP2) without cytosine. We also examined templates containing mononucleotide A, denoted as M-17 (A), which did not undergo mutagenesis. These had a very similar design to the C microsatellite templates detailed in Supplementary Table S1. We use the notation of M-18 (C+) and D-26 (CA+) to denote the templates and libraries after mutagenesis, and M-18 (C−), D-26 (CA−) and M-17 (A−) to refer to libraries without mutagenesis.

### Protocol for partial mutagenesis, library preparation and sequencing

Our operational protocol for partial mutagenesis of microsatellite templates is described here and in Figure 1. In step 1 of the mutagenesis protocol, 80 ng of each of the two templates containing C, M-18 (C) and D-26 (CA), was partially bisulfite converted (or not) by EZ DNA Methylation-Direct Kit (Zymo Research). Incubation time and temperature for bisulfite conversion were chosen to approach an ideal bisulfite conversion rate of close to 50%. In this protocol, DNA was incubated at 55°C for 40–50 min. In fact, we achieved 77% and 66% conversion for the M-18 (C+) tract and the D-26 (CA+) tract, respectively.

After conversion, about 6 × 10^4^ original templates underwent 9 cycles of linear amplification (steps 2 and 3) using a biotinylated oligo. This produced first round copies (first copies, for short) with a structure that had a 5′ biotinylated UP3 and a VT3 represented as ‘NNN…’ in the Supplementary Table S1 and in gray scale in Figure 1. Double-stranded DNA fragments were obtained in another round of linear amplification by using UP1 (step 4). In step 5, extra free oligo was removed by Thermolabile Exonuclease I (NEB). After adding 50 ng of carrier DNA (poly (A), Sigma-Aldrich), biotinylated DNA fragments were purified by streptavidin beads (NEB). In step 6, 18 cycles of the exponential PCR were carried out using UP1 and UP3 to generate enough material to prepare the DNA libraries for sequencing. Standard steps for library preparation (end polishing, A-tailing, adapter ligation) were utilized to complete the sequencing library preparation (step 7). All libraries were prepared with variable length library barcodes (39), and then pooled. The pooled libraries were sequenced as 2 × 150 bp paired-end runs on an Illumina MiSeq™ (step 8).

In steps 3 and 4 (linear amplification of first copies), we used NEBNext^®^ Q5U^®^ Master Mix. This master mix contains modified Q5^®^ High Fidelity DNA Polymerase, optimized for amplification of uracil-containing templates. In step 6 we used Phusion Flash High-Fidelity PCR Master Mix (Thermo Fisher Scientific) for 18 cycles of PCR. This master mix contains Phusion Flash II DNA Polymerase which has high-fidelity and is excellent for multiplex PCR. In step 7, for library preparation, we used NEBNext^®^ Ultra™ II Q5® Master Mix (NEB). This master mix contains Q5^®^ High Fidelity DNA Polymerase, optimized for amplification of NGS libraries.

The parallel protocol without bisulfite treatment, used for the unmutated C templates, M-18 (C) and D-26 (CA) and M-17 (A), had the following differences: the number of original templates was about 3 × 10^4^, and in step 6, 14 or 15 cycles of PCR were employed.

### Sequence processing and tabulation

All read pairs were first evaluated for having the proper structure. A proper read pair has a good match (allowing up to one mismatch) to each of the UP1, UP2 and UP3 regions and the proximal flank of the microsatellite in both reads. From a proper read pair, we can extract the three varietal tags which identify the template (VT1, VT2) and first copy (VT3). From each read of the pair, we also searched for a good match to the distal flank sequence (up to one mismatch), and if the distal flank is found, we reported a microsatellite length (MSL) based on the distance in base pairs (bp) between the flanks within the read. A proper read always has a proximal flank, but it is possible that we could not clearly identify the distal flank. In those cases, the read did not report a length. We say a read pair is qualified if the both paired-end reads agree on the MSL, or if only one read of the pair reports a length. We then label these MSL as on-target if they are the expected length (i.e. 17, 18 or 26), and off-target, otherwise.

For qualified reads, we measured the degree to which the microsatellite is disrupted by the mutagenesis in two ways (disruption indices). The first is the MS conversion rate or the proportion of C bases converted to T in the MS, restricted to the tandem repeat. The second is the maximal residual repeat length, which is the largest number of tandem repeat units present in the disrupted microsatellite. We define a read as sufficiently disrupted if the MS conversion rate is between 0.15 and 0.85 and the maximal residual repeat length does not exceed five. See Supplementary Table S2 for the expected yield of microsatellite disruption as a function of the average bisulfite conversion rate, as determined by simulation. Our observed levels of disruption follow closely the expectations from simulations.

We made a set of 5 tables from the proper reads for each of the five libraries (three templates and two protocols): M-17 (A−), M-18 (C−), D-26 (CA−), M-18 (C+), D-26 (CA+), which we call the READ TABLE (see Data Availability). The READ TABLE records the varietal tag information for the read, the MSL if the read is qualified (−1, otherwise), and its two disruption indices.

From the READ TABLES, we then made the FIRST COPY TABLES. A first copy is marked by its template tag-pair (VT1, VT2) and its first copy tag (VT3). We call a first copy properly-covered if it has a sufficient number of proper reads. The threshold for the number of reads required to define properly-covered depends on the complexity and depth of the library and was 10, 10, 20, 50 and 100 for M-17 (A−), M-18 (C−), D-26 (CA−), M-18 (C+), D-26 (CA+), respectively. The choice for these cutoffs, given the actual distribution of available reads, are shown in Supplementary Figure S1. We call a first-copy well-covered if it is properly-covered and has at least three qualified reads. For each well-covered first copy, we counted the MSL over all qualified reads to determine the modal length, which is the most common length reported by all reads associated with that first copy. A first copy is labeled disrupted if the median disruption parameters over its qualified reads are within the bounds to call a read sufficiently disrupted.

For each first copy, we tabulated the number of proper reads, the number of qualified reads, the modal length, and the number of qualified reads that report the modal length. For each first copy, we also recorded the median disruption indices of its qualified reads, where applicable. We then made the WELL-COVERED FIRST COPY TABLE by restricting to rows with a sufficient number of proper and qualified reads. This filtering step eliminated VT combinations with low read coverage that result from single-base errors in the varietal tag sequences.

From the WELL-COVERED FIRST COPY TABLE, we generated the TEMPLATE TABLE. A template is any template tag-pair (VT1, VT2) with at least one well-covered first copy. For each template, we counted the number of qualified reads, the number of well-covered first copies, and the median disruption indices from its first-copies. If those median disruption indices fall within the criteria defined for a sufficiently disrupted read, we call the template disrupted. We also record the modal lengths of the well-covered first copies for each template. We call a template well-covered if it has at least three well-covered first copies. Well-covered templates are flagged as synthetic variants if three or more first copies unanimously agree on an MSL length different from expected.

### Protocol for biological sample processing with panel enrichment

To evaluate the recovery of microsatellite information from a biological sample, we isolated DNA from a skin fibroblast cell-line (CSHL-SKN1). To enable both linear and exponential amplification after partial mutagenesis, we designed custom fork-tailed adapters such that each of the two tails each contain a different C-free universal primer and distinct random C-free template tags (Supplementary Table S3). We start with 1 μg of genomic DNA, fragmented, end-repaired, 3′ adenylated and then ligated to our custom fish-tailed adapters using the NEBNext^®^ Ultra™ II FS DNA Library Prep Kit (NEB).

As shown in Supplementary Figure S2, we then enrich for DNA fragments containing microsatellites using a custom set of biotinylated probes for either the ‘C-panel’ or the ‘CA-panel’ (xGen™ Custom Hyb Panels, IDT DNA). Each panel includes 1260 oligonucleotides in 630 pairs. Each pair comprise two 60-bp biotinylated oligonucleotides complementary to the flanking sequence of a microsatellite in the human genome. For the C-panel, microsatellite targets were mono-nucleotide C while the CA panel comprised dinucleotide CA repeats. Criteria for selection of appropriate targets include the length of the MS in the reference genome and the uniqueness of the flanking sequence in the human genome. Sequences for the capture oligonucleotides are included in Supplementary Table S4.

After enrichment, DNA fragments were partially bisulfite converted (or not, for the control samples) using conditions as described above for the synthetic templates. Steps for library amplification and adaption for sequencing are also as above, with the following modifications: we performed 30 cycles of linear amplification and included a step to select for DNA fragments in the range of 300–600 bp. This was followed by a final PCR amplification with library specific sequencing primers. Sequencing was done on the NextSeq 500 in 2 × 150 bp run mode (Illumina).

### Data processing for panel enriched samples

Raw sequence reads are disassembled to extract varietal tags and primer sequences. The residual bases are then mapped to the human genome (hg38) using a pipeline similar to those used for standard bisulfite sequence mapping: each read-pair is mapped in two versions: (i) with C-to-T conversion on read 1 and G-to-A conversion on read 2 and (ii) with G-to-A conversion on read 1 and C-to-T conversion on read 2. Both conversion cases are mapped to each of two genomes: (A) hg38 under C-to-T conversion and (B) hg38 under G-to-A conversion. The result is four distinct mappings for each read-pair and we select the best map. By library construction, the best maps are almost always 2A and 2B where the choice of genome is often a function of which converted strand was amplified. Mapping is performed using Bowtie2 (40).

Mapped reads that localize to a target microsatellite locus are tested for whether the fragment spans the microsatellite locus. We check if the read-pair maps across the locus and also if either read contains the 20 bp sequences flanking the microsatellite (up to C-to-T conversion). If either single read in the pair contains both the left and right flanks of the microsatellite, we record the distance between the flanks as the length of the microsatellite. The intervening sequence is also measured for its conversion rate and the length of its maximal residual repeat. We used this mapping method to analyze coverage, on-target capture rates and spanning ratios.

When analyzing panel data, we are interested in only a particular subset of genomic positions. We can significantly improve the speed of alignment by restricting the target sequence to just those regions. To this end, we developed a custom suffix array based on C-to-T conversion in the targeted genomic strand for a region of 1000 bp surrounding each of the 1260 panel loci. The purpose the first-round mapping is to localize the reads for subsequent flank search so that off-target maps will not contribute to false counts. At present, we confine our measurement of disruption to the microsatellite itself. For that reason, we exclude 284 loci (242 C loci and 42 CA loci) with repeats of 5 of more units in either of the 20 bp flanks.

## RESULTS

### Experimental design

In the first part of the Results, we evaluate the performance of the partial mutagenesis protocol for microsatellite length determination in the well-controlled setting of synthetic templates. Figure 1 shows our protocol for library preparation from synthetic templates. The templates have microsatellite tracts flanked on either side by common sequence (black). 5′ and 3′ to the common sequence are varietal tag sequences (VT1 and VT2, blue) that uniquely label each template molecule. Flanking the varietal tags are universal primer sequences (UP1 and UP2′, green) designed without C nucleotides to resist bisulfite mutagenesis. These templates were either bisulfite treated or not, in step 1. A biotinylated primer complementary to the 3′ universal primer UP2′, with its own unique varietal tag (VT3, gray) and a universal primer sequence (UP3) is added in step 2. We generate multiple first copies (step 3), each with the same VT1-VT2 and a unique first copy tag VT3. The first copies are made double-stranded (step 4), purified by streptavidin chromatography (step 5), and amplified by PCR (step 6). Finally, the PCR products are made into libraries with specific barcodes (step 7). In the second part of the Results, we utilize biological templates from a cell line, which were obtained from sheared genomic DNA, end-adapted, and enriched using panels designed to capture sequences flanking microsatellites. Thereafter, a similar protocol was followed. We focus our experiments primarily on two types of microsatellites containing cytosines: mononucleotide repeats of Cs and dinucleotide repeats of CA. Other C containing repeats are potentially amenable to the bisulfite partial mutagenesis approach, albeit with some added complexities: partial mutagenesis of CT repeats can introduce new mononucleotide repeats, and mutagenesis of CG repeats may be stifled by methylated cytosines common in the CG repeat context.

### Measuring MSL from disrupted and undisrupted synthetic templates

We ordered three synthetic templates, one with a tract of 17 A, one with 18 C, and one with 13 CA repeats. The two with C were treated or not with bisulfite, resulting in five libraries named M-17 (A−), M-18 (C−), D-26 (CA−), M-18 (C+), D-26 (CA+), corresponding to M (mono-) or D (di-nucleotide), their microsatellite length, the sequence of the microsatellite repeat unit, and whether mutagenesis was applied (+/−). The datasets from these libraries are named similarly, but sometimes abbreviated to A−, C−, CA−, C+ and CA+, respectively. A template is disrupted if the mutagenesis produces copies with a reduced tandem repeat structure, which we can condense to the maximal residual repeat or MRR (see Materials and Methods). In the analyses below, when we restrict the dataset of the mutated libraries to only the sufficiently disrupted templates, we refer to these datasets as M-18 (C++) or C++, and D-26 (CA++) or CA++. Given our conversion rates, the proportion of sufficiently disrupted reads in the C++ and CA++ libraries is 29% and 73%, respectively, which falls close to the expected proportion (see Supplementary Table S2).

Below we describe the properties of the microsatellite lengths observed in the datasets at three levels of organization: (i) reads, (ii) first copies and (iii) templates. Templates are uniquely identified by their VT1-VT2 tag-pair. First copies, which are generated during the first rounds of linear amplification (Figure 1, step 2), are identified by the unique triplet: the VT1–VT2 pair from their initial template, and the unique VT3 added to the molecule during linear amplification. For each read-pair with the correct structure we determine its three varietal tags. Within a single read, we measure the microsatellite length (MSL) from the distance between the expected proximal and distal flank sequences, if both are observed.

#### Reads

For the M-18 (C−) library, we found that the base quality of the read decays considerably after reading through the microsatellite sequence (Supplementary Figure S3), consistent with known issues sequencing through repeat sequences (20,21). In many cases, this decay of base quality is so bad that the distal flank sequence could not be identified in the read. In the M-18 (C−) dataset, only 46% of read-pairs report a consistent length. In contrast, for the M-17 (A−) 95% of read-pairs report a consistent length, and for the remaining sets the rate exceeds 99% (Supplementary Figure S4).

**Figure 2.**
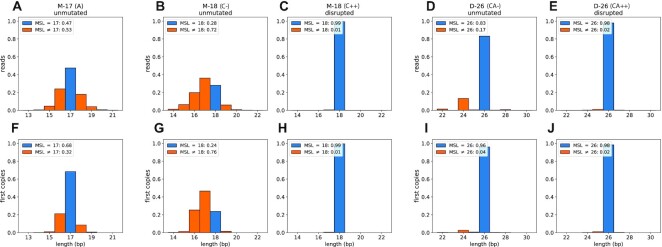
MSL distributions for reads and first copies. For each of the five libraries, we plot the distribution of observed MSLV. The upper panels show the distribution of read counts and the lower panels show the distribution of first copy modal lengths. The expected length is shown in blue, with off-target lengths shown in orange. The plot legends summarize the on and off-target rates per library. Data used to generate these plots is included in Table 1.

In Figure 2, panels A–E, we show the microsatellite length determinations per read as a histogram for each of the five libraries. In the histogram, reads that match the expected length are shown in blue (on-target), while those reporting a different length are shown in orange (off-target). In general, off-target lengths tend to be shorter rather than longer, consistent with a deleterious bias during replication (30). Read counts for each MS length and library are shown in Table 1A.

**Table 1.** MS length distributions in reads and first copies

**A. READS**			**MSLV deviation from expected length**										
			**<= −5**	**−4**	**−3**	**−2**	**−1**	**0**	**+1**	**+2**	**+3**	**+4**	**>= +5**
M-17 (A−)	unmutated	all	1540	2382	15 687	129 032	662 810	1 318 654	496 859	114 349	19 831	3342	5390
M-18 (C−)	unmutated	all	4913	2511	10 068	31 175	56 529	44 531	9785	1360	229	51	200
M-18 (C++)	disrupted	all	1072	1	5	14	8451	1 641 719	60	2	0	0	0
D-26 (CA−)	unmutated	all	8957	37 395	2357	339 586	14 455	2 120 035	5291	18 719	18	702	121
D-26 (CA++)	disrupted	all	8395	206	848	2544	51425	4 413 601	14353	244	1	10	3
M-18 (C++)	disrupted	wc - synth	2	1	5	12	509	1 612 051	54	2	0	0	0
D-26 (CA−)	unmutated	wc - synth	3777	32367	413	303560	1321	1 910 332	450	16 818	0	589	115
D-26 (CA++)	disrupted	wc - synth	61	196	2	2453	2076	4 312 537	405	236	0	10	3
**B. FIRST COPIES**			**MSLV deviation from expected length**										
			**<= −5**	**−4**	**−3**	**−2**	**−1**	**0**	**+1**	**+2**	**+3**	**+4**	**>= +5**
M-17 (A−)	unmutated	all	96	58	180	2250	50 762	164 225	20 588	1515	112	13	5
M-18 (C−)	unmutated	all	294	81	383	7171	13 146	6717	428	11	0	0	4
M-18 (C++)	disrupted	all	7	0	0	0	58	11 644	0	0	0	0	0
D-26 (CA−)	unmutated	all	86	82	23	1211	275	44 142	95	36	0	2	1
D-26 (CA++)	disrupted	all	13	0	2	0	61	5852	18	0	0	0	0
M-18 (C++)	disrupted	wc - synth	0	0	0	0	0	11 415	0	0	0	0	0
D-26 (CA−)	unmutated	wc - synth	6	54	5	1047	25	39 557	8	32	0	1	1
D-26 (CA++)	disrupted	wc - synth	0	0	0	0	0	5680	0	0	0	0	0

For each of the five libraries, we show the number of reads and number of first copies that show each possible microsatellite length, from < = −5 to > = +5 from the expected length. For the three most stable libraries, where we were able to identify and remove synthetic variants, we show the resulting counts after removing synthetic variants.

For M-17 (A−) only 47% of the reads report the expected length of 17 bp. For M-18 (C−) the results are even worse: 28% of reads report the expected length. In contrast, the M-18 (C++) have 99% of reads on-target. For the D-26 (CA−), we find 83% of reads report the on-target length, and the most frequently reported off-target variants occur at 2 bp increments, equivalent to the size of the repeat unit. In contrast, the disrupted D-26 (CA++) templates have a high on-target rate with 98% reporting a length of 26. Unlike the unmutated D-26 (CA−), the off-target variants in D-26 (CA+) are almost entirely 1 bp off, reporting lengths of 25 or 27, suggesting they may result from error during synthesis, which we call synthetic variants.

#### First copies

We can, in principle, improve accuracy by taking a consensus of lengths from reads over first copies. This improvement in accuracy will be most effective when error rates are well below 50%. We define the modal length of a first copy as the value most commonly seen among all the reads associated with it. The distributions of modal lengths are displayed in Figure 2, in panels F–J, and reported as counts in Table 1B. For the M-17 (A−) library, we see a slight improvement of the proportion of MS lengths on-target, from 47% when counting reads to 68% when counting the first copy consensus. For the M-18 (C−) library, we see a decline in lengths on-target from 28% to 24%. As expected, the unmutated A− and C− libraries do not see significant improvement in accuracy from first copy consensus, due to their high error rates. In fact, the C- error rates are so high that first copy consensus actually *increases* the error rate. In contrast, MS length estimates from the D-26 (CA−) unmutated library improve significantly when based on first copies, with 96% on-target, compared to 83% for reads alone. Unexpectedly, the lengths based on disrupted MS have nearly identical on-target rates, about 98% for D-26 (CA++) and 99% for M-18 (C++), whether using reads or first copy consensus. As we will now show, the small number of off-target lengths in the original synthetic templates arise from synthetic variants. We focus our analysis of synthetic variants and error rates on M-18 (C++), D-26 (CA−), and D-26 (CA++) where the error rates are most amenable to consensus calling.

#### Detecting synthetic variants

**Figure 3.**
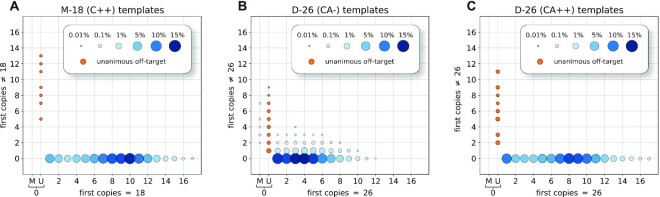
First copy consensus per template. For three libraries, we plot the distribution of templates with *x* on-target first copies and *y* off-target first copies. The size of the dot and intensity of the color reflect the proportion of templates, normalized by the total template count. Templates with no on-target first copies were further divided into ‘U’ if all the first copies were unanimous for the same off-target length, or ‘M’ if the first copies lengths were mixed. To highlight the population, unanimous off-target template populations are shown in orange. The underlying count data are available in Supplementary Table S5.

For each initial template, we tabulate the modal lengths over all of its first copies. We condense this information by counting the number of first copies on-target (*x*) and the number of first copies that are off-target (*y*). Figure 3 shows a scatter plot summarizing the distribution of (*x*, *y*) over all templates for each of the three libraries: M-18 (C++), D-26 (CA−) and D-26 (CA++). The size of the dot and intensity of the color reflect the proportion of templates with those values. We split templates with zero on-target first copies into those whose first copies are unanimous for an off-target length (orange, column U) and those whose first copies show multiple off-target lengths (blue, column M). The data underlying the figure are found in Supplementary Table S5.

The templates with disrupted MS, both the mono- and di-nucleotide repeats, fall into two cleanly separable groups: the vast majority, in which the consensus MS length of first copies all unanimously agree with the on-target length; and a much smaller number of outlier templates, in which none of their first copies have an on-target length. All outlier disrupted templates were further examined. For each of these, all first copies unanimously agree on their unexpected MS length (shown in Supplementary Table S6), most commonly one base-pair less than the expected length. This confirms the hypothesis of the synthetic variants, and demonstrates a method for their detection in disrupted data.

#### Estimating error in the absence of synthetic variants

By removing the synthetic variants we can get a clearer picture of error rate in the disrupted templates. To make a fair comparison, we also remove synthetic variants from the unmutated D-26 (CA−) data, but extra care is required because of its higher error rate. As seen in Figure 3B, the aggregate over templates for D-26 (CA−) shows a complex pattern. A majority of templates are on-target, with all first copies in unanimous agreement. There is also a small proportion of outlier templates, unanimous in that no first copies have the on-target length. Most, but not all, of these are unanimous for another length, typically one base less than the expected length (see Supplementary Table S6), and are flagged as synthetic variants. The third and fairly numerous group of templates in D-26 (CA−) have mismatched first copies, typically disagreeing by units of the repeat length. These we attribute to read error, and do not remove.

We estimate MS length read error from these three datasets and as shown in Table 1A. The read error rates for disrupted reads in the M-18 (C++) and D-26 (CA++) data are on the order of 10^–3^ or better, but remain at about 16% for the undisrupted D-26 (CA−) data. Almost all of the erroneous reads in the D-26 (CA−) have MS lengths of 24. The erroneous reads for D-26 (CA++) are almost equally divided between lengths 24 and 25 (−2 and −1). The former value probably arises from residual tandem repeats following disruption. Consistent with this, we examined error rates as a function of disruption parameters (Supplementary Table S7), and note that if we had been even more restrictive, the read error rates could be reduced further.

In Table 1B, we show the error in first copy consensus length, before and after removal for synthetic variants. After removal, we have 11 415 and 5680 first copies in the M-18 (C++) and D-26 (CA++) data, respectively, all of which are of the expected length. Thus the error in the first round of replication is on the order of 0.5 in 17 095 or about 3 × 10^–5^ or less. First copy error for the CA− is reduced somewhat, but still stands at about 3 × 10^–2^. Thus, disruption lowers the per round error rate by three orders of magnitude. By modeling a PCR amplification with a known number of rounds, we arrive at similar per round error rates, 2.8 × 10^–5^, 4.95 × 10^–5^, and 1.35 × 10^–2^ for C++, CA++ and CA−, respectively (41).

### Detection and estimation from matched read data

In the previous sections, our experimental design encompassed synthetic templates that (i) almost all contain the same microsatellite length and (ii) have high read coverage over (iii) many first copies for each template. We used this information to confidently detect and then remove synthetic templates of variant length before measuring read and per round error rates. In most practical applications, however, each of these three conditions will be absent. In a biological sample, there will often be multiple true lengths and, depending on the assay, the depth of coverage may include only a few reads per template.

In this section, we introduce a simple method for estimating error from sparsely covered data from templates with many variant lengths. This method uses independent *k*-multiplets: counts of microsatellite lengths from *k* different first copies of the same template. We first demonstrate this method on our synthetic templates before applying it to a biological sample. We will show that, without removing synthetic variants and with as few as two or three independent reads, this method accurately estimates error rates and can detect synthetic variants with high confidence from disrupted templates. As before, we apply this approach to M-18 (C++), D-26 (CA−) and D-26 (CA++), where error rates are well below 50%. For situations where the error exceeds 50%, these estimates are unreliable. We also note that this method approximates the total error irrespective of directionality and so serves as an upper bound for any particular type of error.

**Figure 4.**
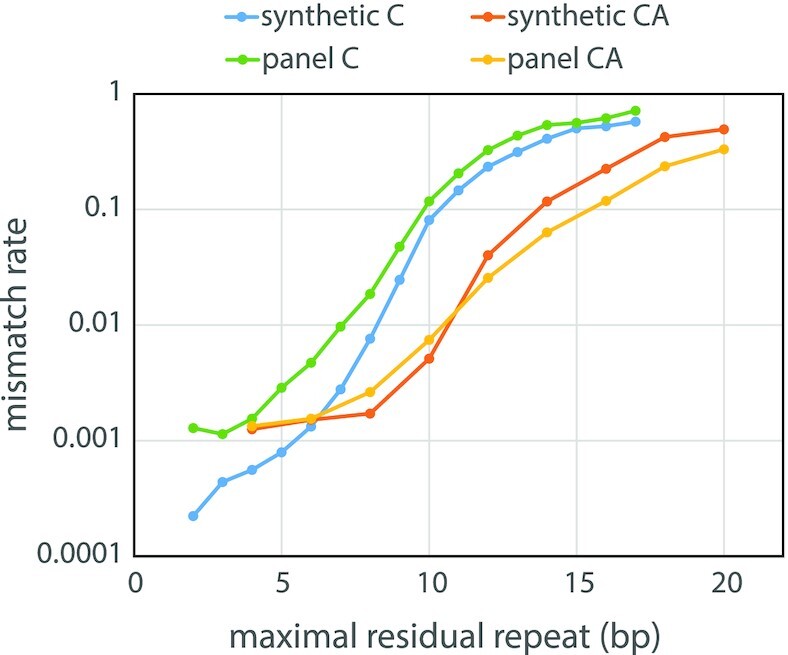
Mismatch rate as a function of maximal residual repeat. By measuring the rate at which pairs of reads from different first copies disagree, we can approximate the error rate in measuring a microsatellite length. Here, we examine the effect of the maximal residual repeat length on the rate at which pairs of independent reads from the same template disagree. We perform this calculation over synthetic templates and biological templates, separately for mono-C and di-CA templates.

We first obtain an estimate of error rates by examining independent doublets (2-multiplets): pairs of lengths from two different first copies. For each pair of doublets that disagree on length, we assume that one of the two reads is in error. If the chance that one read is in error is *p*, then the probability of observing a mismatch is *x* = 2*p*(1 – *p*). After measuring the mismatch rate over doublets, *x*, we invert this formula to approximate the error rate, *p* = $\frac{1}{2}( {1 - \ \sqrt {1 - 2x} } )$. In Figure 4, we show the mismatch rate for the C+ and CA+ synthetic libraries over a wide range of maximal residual repeat (MRR) values. For the C repeats, the mismatch rate increases exponentially as the MRR increases from 5 to 10 bases in length. For the CA repeats, the mismatch rate increases exponentially as the MRR increases from 8 to 14 bases in length (4–7 repeats). Aggregating over all templates with an MRR of 5 or less (as per our definition of disruption), we calculate an average mismatch rate of 6.68 × 10^–4^ for C++ templates and 2.52 × 10^–3^ for CA++ templates. Solving for *p* above, the estimated error rate is 3.34 × 10^–4^ for C++ and 1.26 × 10^–3^ for CA++. These values nearly coincide with the read error rate estimates in Table 1A after removal of synthetic variants: 3.63 × 10^–4^ and 1.26 × 10^−3^ for C++ and CA++ respectively.

To count over *k*-multiplets in the synthetic data, we partition the first copies of each template into distinct subsets of size *k*. The number of such subsets is given in Table 2 (number of *k*-subsets). For each *k*-subset, we determine all possible *k*-multiplets, drawing a unique read from each of the first copies. The average rate at which the *k*-multiplets were in unanimous agreement is shown in Table 2 (observed unanimity rate). For each *k*-subset, we also determine the most common unanimous *k*-multiplet. We then measure the proportion of unanimous *k*-multiplets that differed from this length. The average rate of conflicted unanimous lengths is shown in Table 2 (unanimity conflicts).

**Table 2.** *k*-multiplet observed and predicted from synthetic templates

Library	*k*	Number of k-subsets	Number of k-multiplets	Observed unanimity rate	Observed unanimity conflicts	Theoretical unanimity rate	Theoretical unanimity conflicts
**CA−**	**2**	21 062	6.09E+07	0.7281	2.36E−02	0.7312	3.50E−02
**CA−**	**3**	12 110	1.89E+09	0.6024	6.50E−03	0.5968	6.86E−03
**CA−**	**4**	7682	6.68E+10	0.5082	1.99E−03	0.4985	1.31E−03
**CA−**	**5**	5048	2.40E+12	0.4271	3.97E−04	0.4183	2.51E−04
**CA++**	**2**	2795	1.56E+09	0.9974	1.58E−06	0.9975	1.59E−06
**CA++**	**3**	1722	7.36E+11	0.9961	4.58E−09	0.9962	2.01E−09
**CA++**	**4**	1192	3.84E+14	0.9949	1.53E−11	0.9950	2.53E−12
**CA++**	**5**	878	2.14E+17	0.9937	1.04E−13	0.9937	3.20E−15
**C++**	**2**	6082	1.05E+08	0.9993	1.02E−07	0.9993	1.32E−07
**C++**	**3**	3815	8.63E+09	0.9990	0.00E+00	0.9989	4.79E−11
**C++**	**4**	2667	8.05E+11	0.9986	0.00E+00	0.9985	1.74E−14
**C++**	**5**	2016	7.83E+13	0.9982	0.00E+00	0.9982	6.31E−18

For the three libraries (CA−, CA++ and C++) and for values of *k* between two and five, we randomly partition the first copies of each template in non-overlapping subsets of size *k*, the number of which is reported in the column ‘number of *k*-subsets.’ For each such *k*-subset, we consider all possible *k*-multiplets the total count of which is reported in the column ‘number of *k*-multiplets.’ The rate at which *k*-multiplets are unanimous for microsatellite length appears in the column ‘observed unanimity rate.’ Each *k*-subset has a majority unanimous length; the average proportion of unanimous k-multiplets that do not agree with that length appears in column ‘observed unanimity conflicts.’ The theoretical predictions for unanimity rate and unanimity conflict appear in the last two columns.

Assuming the error rate *p* as determined above were a constant for each first copy from every template, we make theoretical estimates for both the unanimity rate and the rate of unanimity conflict. We can estimate the probability of observing a *k*-multiplet in unanimous agreement by the formula *p^k^* + (1–*p*)*^k^* (Table 2, theoretical unanimity rate). Likewise, the estimate for unanimity conflicts is given by the formula *p^k^ / (p^k^* + (1–*p*)*^k^*) in Table 2 (theoretical unanimity conflicts). The observed and theoretical results are in excellent agreement.

We observe as follows. As *k* increases, the probability of unanimous agreement decreases. However, the yield of unanimous *k*-multiplets from disrupted templates is nearly perfect: even five reads occur in agreement better than 99.3% and 99.8% from CA++ and C++, respectively. In contrast, only 42.7% of the time does the CA− library yield unanimous agreement between five first copies. Also, as *k* increases, the chance that a unanimous result is in error declines. For the unmutated CA- library, 2-multiplets are unanimous, and in error, 2 in 100 times (∼0.16^2^). Requiring five first copies in unanimous agreement reduces that error rate to ∼4 in 10 000. In contrast, the disrupted CA++ and C++ data have 2-multiplet unanimous error rates that are 1 in 500 000 and 1 in 10 000 000 respectively. Synthetic variants behave the same as on-target synthetic templates (Supplementary Table S8A). The synthetic variants we detect this way are precisely the templates identified by our previous methods.

### Measuring MSL at microsatellite loci

We apply our methods to measuring microsatellite lengths at select loci in the genomic DNA of a human cell line (see M&M). We use biotinylated hybridization probes to enrich for microsatellite loci and purchased a panel of 1260 paired capture oligonucleotides that flank microsatellites, chosen to be relatively unique within the human genome (Supplementary Table S4). 630 pairs flank loci containing a mononucleotide C repeat, and 630 flank a dinucleotide CA repeat. We fragmented genomic DNA, ligated to varietal tags, captured with our panels, then subjected the enriched sequences to partial bisulfite conversion and multiple rounds of tagged linear amplification. After sequencing, reads were deconvoluted by template and first copy tags, and aligned to the human genome using standard techniques for mapping bisulfite sequence data. Enrichment was highly successful: >72% of reads were on target, achieving better than 2000-fold enrichment with excellent uniformity of coverage across loci (Supplementary Figure S5).

#### Improving sequence quality with disruption

To measure error rates by mismatch, we must first determine microsatellite lengths from reads. This requires observing matching sequence for 20 bp flanking the microsatellite on both ends of the same read. For undisrupted mononucleotides, this is highly constrained by the poor sequence quality following a long repeat. We quantify the quality of the sequence as follows.

We say that a read-pair brackets a microsatellite locus if sequences from both sides of that microsatellite can be read. We say that a read-pair covers a microsatellite if at least one read of the read-pair contains the microsatellite and 20 bp of both flanks. For each locus, we record the spanning ratio of read-pairs covering to bracketing the microsatellite. The expectation is that a low ratio reflects poor read quality caused by the microsatellite. The spanning ratio depends strongly on whether the library was mutagenized or not. Additionally, the ratio varies by the composition and length of the microsatellite loci.

**Figure 5.**
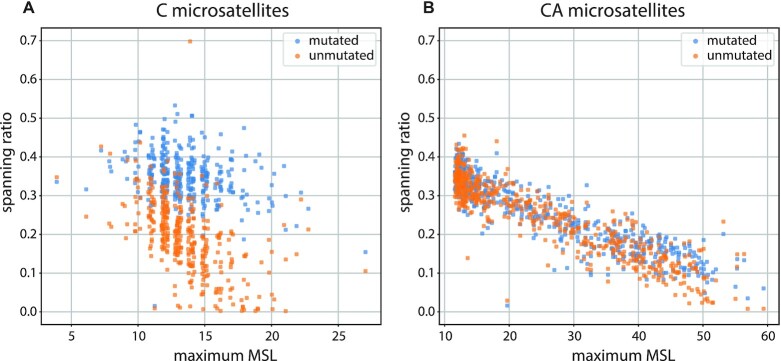
Spanning ratio as a function of microsatellite length. For each locus in the panel, we define the spanning ratio as the rate at which read-pairs that brackets the microsatellite contain a read that covers the microsatellite. Each locus was assigned a length (or the longer of the two allele lengths, in the case of heterozygous loci) from the disrupted data, indicated on the X-axis, with the spanning ratio on the Y-axis. The unmutated libraries are shown in orange and the mutated libraries are shown in blue. Panel **A** shows the mono-C loci and panel **B** shows the di-CA loci.

Figure 5A, B shows the spanning ratio for the mutagenized and unmutagenized libraries for mono-C and di-CA microsatellites, respectively, aggregated by locus. Each locus was assigned a length (or the longer of the two allele lengths, in the case of heterozygous loci) from the disrupted data, indicated on the X-axis, with the spanning ratio on the Y-axis. The spanning ratios are somewhat similar for the mutagenized or unmutagenized CA microsatellites, consistent with the tolerance of the sequencer for CA repeats. Nevertheless, we see improvement in the spanning ratio with mutagenesis when the CA tract is very long. Mutagenized or not, we see a decline in ratio as the length increases, consistent with the inherent difficulty in covering longer microsatellites within a 100 bp read. Spanning ratios for mono-C loci behave very differently. Whereas the mutagenized dataset maintains a spanning ratio of about 0.4 across the whole range of lengths, the spanning ratio for the unmutagenized data drops considerably after 10 Cs. This is in keeping with the poor quality of the synthetic mono-C template (Supplementary Figure S3).

#### Measuring mismatch

As discussed in the previous Matched Read section, we examine reads from the same template but different first copies. The proportion of lengths that mismatch is a function of error rate. As we have seen in the first part of this paper, the error rate depends on template class properties: mono- or di-nucleotide and maximal residual repeat length. We compute the average mismatch rate over templates, aggregated by class (CA and C, synthetic and biological), as a function of maximal residual repeat, shown in Figure 4. We observe that the templates with shorter maximal residual repeat lengths have lower mismatch rates. This is true for synthetic and biological templates, whether composed of mono- or dinucleotide repeats. The curves for synthetic templates overlap the curves for biological templates, from which we may safely infer that error rates are virtually the same for synthetic and biological templates.

In the Supplementary Table S8B, we show both the observations and theoretical predictions for the biological template data, following the format of Table 2. We estimate first copy read error rates as we did in the previous section. As can be seen, the theory which worked so well for synthetic templates works as well for biological templates.

#### Improved MSLV profiling with disruption

To demonstrate the power of mutagenesis, we compare the profiling of microsatellite lengths in the panel loci made from unmutated and disrupted panel libraries. For this purpose, we chose 3-multiplets. For each biological template with at least three distinct first copies, we select a single 3-multiplet; that is, we draw a single read from each of three randomly selected first copies. We have fewer triplets from the unmutated panel libraries than the disrupted panel libraries, so we down-sampled the disrupted triplets to obtain equal levels of coverage (33K triplets from the mono-C loci, 140K from the di-CA loci).

**Figure 6.**
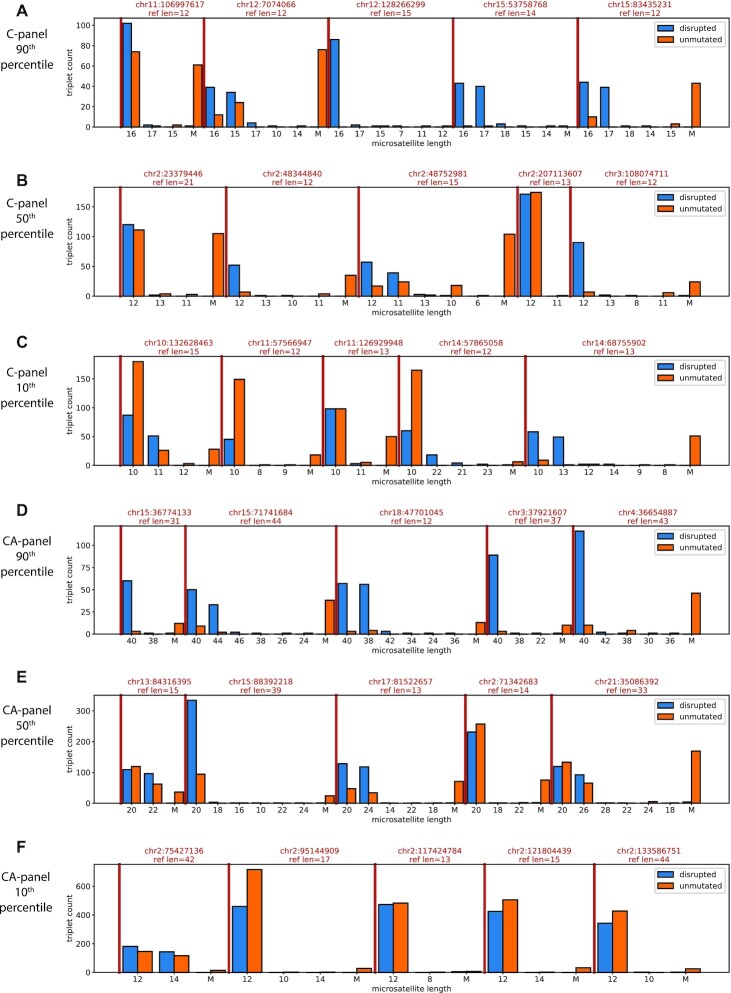
Representative triplet length observations from cell line sample. We sampled the same number of triplet observations (3 reads from independent first copies) from disrupted and unmutated libraries from 388 mono-C and 588 di-CA microsatellite loci separately. For each locus, we count the number of unanimous triplets by their reported length and count the number of triplets that have a disagreement (‘M’). Within each locus, lengths are sorted by their count in the disrupted data but data are shown for both disrupted (blue) and unmutated (orange) libraries, with ‘M’ always appearing last. The loci are also sorted by their most common length in the disrupted data. In panel **A**, we show five mono-C loci where the major length is in the 90th percentile. For these five loci, the major length is 16 bp. Red lines separate loci which are also labeled with their position in hg38 and their length in the reference genome. In panels **B** and **C**, we show five mono-C loci at the 50th percentile and the 10th percentile for major length respectively. Panels D-F show the 90th, 50th and 10th percentiles for major length for the CA-loci.

When the triplet reads are in unanimous agreement, we report the consensus length; otherwise we set the length to a value of ‘M’ for mismatch. For each genomic locus we tally the number of triplets reporting each length (Supplementary Table S9). Segregating loci by motif (C and CA), excluding loci with less than 20 templates, and then sorting loci by their primary length (the most common disrupted template length), we can compare the observations between disrupted and unmutated templates. In Figure 6, the first three panels show mono-C microsatellite loci. In panel A, we show five microsatellite loci with primary length 16 (at the 90-th percentile for primary length); in panel B, five loci with primary length 12 (at the 50th percentile for primary length); and in panel C, five loci with primary length 10 (at the 10th percentile for primary length). The next three panels show di-CA microsatellite loci, also at the 10-th, 50-th and 90-th percentiles for primary length, respectively.

For the mono-C data, we observe good unanimous coverage from both disrupted and unmutated templates only when the microsatellite length is short: 10–12 bp or less (i.e. panel C). However, there is poor yield of triplets from unmutated templates as their length increases, and few are unanimous (panels A, B). There is a similar story in the di-CA data. We observe good unanimous coverage for disrupted and unmutated loci when the lengths are short (12 bp, panel F). However, at the loci with longest lengths many alleles are unreported from the unmutated data (panel D), and even at moderate lengths (panel E) the unmutated templates show many mismatches. Multiplets from disrupted templates are almost never mismatched.

## DISCUSSION

The length of a microsatellite is unstable during replication. On the one hand, their high rate of variability makes microsatellite lengths attractive markers for disease; on the other hand, instability makes accurate microsatellite length measurement very difficult (16–19). This is especially true for the longer mononucleotide repeats, which are the most highly variable microsatellites and potentially the most valuable markers. The measurement of mononucleotide repeat length is further aggravated by modern high-throughput sequencing platforms (20,21,42). As we discuss in the Introduction, various attempts have been made to ameliorate some of these problems (8,22–24). However, none of these approaches have the breadth, depth and sensitivity to detect minor populations of variant lengths over many thousands of templates and loci in a single assay.

We solved this problem by conceiving and implementing the strategy of measuring microsatellite length in templates in which we first disrupted the very structure rendering their replication unstable. We disrupted the templates using bisulfite, which converts C to a U, later read as a T. Partial mutagenesis is required, as when the mutagenesis is too complete, a new repeat structure is created, and replication again becomes unreliable. Thus, our method depends on determining the degree of disruption of the repeat structure in each sequence read. We developed two disruption indices, and used them both to identify templates with lower replication error rates.

To demonstrate the ability of partial mutagenesis to stabilize microsatellites for amplification and sequencing, we created a controlled test system using synthetic templates. Each template was synthesized with an identifying tag, and we made multiple independent first copies, each with its own identifying tag. We determined the fidelity of replication as a function of the degree of disruption of the repeat structure. By aggregating over the individual first copies and the individual initial templates with extremely high depth of coverage, we were able to detect templates that were synthetic variants, and then to determine with great precision the error rate in microsatellite length measurement.

We obtain two to three orders of magnitude decrease in per round error rate when the repeat structure is disrupted. The greatest improvement is with mono-C tracts which not only have very high error rates, but often fail to generate a robust read. Upon partial disruption error rates in length measurement of a template are less than 10^–6^ if we observe consensus with even just two reads, each from different first copies.

Using this observation, we developed a method based on two reads per template from first copies (i.e. independent doublets) to estimate error rates and correctly identify variants. We confirmed this method with synthetic templates, and then applied it to biologically derived samples, namely microsatellite loci enriched by panels from a cell line. We showed error rates in length measurement that matched our expectations from the synthetic templates. Disruption of the microsatellite reduces error dramatically, allowing for the simplified error model introduced here to fit the observed data. However, future work could incorporate additional features, such as length-dependent error rates and bias to deletions, as noted by others (25,28–31).

Highly accurate and quantitative MSLV determination has implications for numerous fields of study. Population-level genotyping of healthy and disease-focused cohorts are presently limited by existing approaches for MS measurement. Our understanding of disease-associated microsatellites such as those linked to Huntington's disease (14), fragile X syndrome (15), myotonic dystrophy (43), and other neurological disorders (44) may improve as MSLV accuracy improves. MSLV also has tremendous potential as a marker for single cells and clonal expansions of cells, enabling cell linage tracing to resolve questions in somatic phylogeny (9,10).

Our work has been primarily motivated by the many applications of MSLV in cancer (3–6). These include (a) quantitative microsatellite instability (MSI) assessment, (b) measuring the load of a cancer with known genomic variation; and (c) detecting cancer early in persons at risk. These questions require the high degree of sensitivity and accuracy afforded by the present method.

An extreme level of MSI is itself a condition in some cancers, due to defects in DNA-repair pathways (1,2). For this reason, measurement of the length instability is used clinically to direct treatment (7,23,24,27,45). Better MSLV sensitivity and accuracy could allow for MSI profiling with a more quantitative lens that may improve basic understanding of tumor evolution and prognostication.

The second goal of non-invasive measurement of tumor burden has been achieved using patient specific single nucleotide variations (SNVs) (35,46–50). But while SNVs are sparse and can occur anywhere in the genome, MSLV are frequent and occur at known loci. Therefore, given enrichment for those loci with panels and a reliable method for measuring length, an assay based on microsatellites would be much less expensive than the alternatives, standard for every patient, and could be more rapidly deployed. Moreover, an assay based on a panel of many variable loci might well detect the emergence of a new variant that potentially indicates the escape of a new clone from therapeutic control.

A transformative value for MSLV detection may lie in its application to early detection. Many studies, including our own unpublished work, indicate that upon presentation with cancer most if not all patients have traces of their cancer genome in the cell free component of blood (46,49,51). It follows that detection of neoplasm in the blood is a path to early detection of cancer. While this could in principle be done using SNVs from panels of driver genes (51) or with deep whole genome sequencing of cell free DNA (52), the first will miss many cases and the second would be prohibitively expensive.

To carry out an MSLV assay, as we showed in our cell line pilot studies, DNA templates from clinical samples would be tagged, enriched for selected loci with panels, subjected to mutagenesis, replicated with independently tagged first copies, and then amplified for sequencing. In such assays, one could add synthetic control templates with known microsatellite lengths to provide a measure of error rates, so that the limits of detection could be modeled, as we showed in this paper. We can identify some uncertainties in this plan. (i) Is there sufficient MSLV in tumors? (ii) Is there too much instability in a tumor to get a clear read-out? (iii) Is there too much somatic variation in blood for tumor signal to be seen? We have now the tools to answer these questions.

We can also anticipate some answers. First, while there are hundreds of thousands of microsatellites in the genome, assays based on only a few thousand might easily suffice for cancer detection. We estimate from the literature, and from our own internal tumor sequence data, that at least 1% of mononucleotide tracts have length variation in primary cancers (3–6), much higher of course in patients with MS instability (MSI) syndromes (1,2). We think it highly likely that this is an underestimate, as we have found in the work shown here that many of the mononucleotide C tracks, especially the longer ones, cannot be correctly read or even covered by standard sequencing libraries and platforms. Moreover, panels can be adjusted to increase the representation of highly variable loci. Even if the variation were only 1%, sufficient numbers of variant markers would be present after enrichment of a few thousand loci. Second, if the mononucleotide tracks are too variable in a given cancer, for example in patients with high levels of MSI, the panels can be designed to include more stable microsatellites, such as dinucleotide tracts, or shorter tracts of mononucleotides. Third, while we expect to see some somatic variation in cell-free DNA in healthy individuals, we expect those variant length profiles will be relatively stable over time and distinct from the emerging new variants of a neoplasm.

## DATA AVAILABILITY

Sequence data is available in the Sequence Read Archive, BioProject accession number PRJNA763883. Processed data tables for synthetic data (read, well-covered first copy, and template tables) are available in Zenodo, https://doi.org/10.5281/zenodo.5149074. See Supplement for panel-enriched processed data tables.

## Supplementary Material

gkac723_Supplemental_Files
