# Supplementary material for: Accurate measurement of microsatellite length by disrupting its tandem repeat structure
Source: Nucleic Acids Res. 2022 Sep 12;50(20):e116. doi: 10.1093/nar/gkac723 (PMC9723644; doi:10.1093/nar/gkac723)
Supplement: gkac723_Supplemental_Files [file gkac723_supplemental_files.zip › Supplementary PDF - legends and figures.pdf]

Supplementary Figures and Tables

Supplementary Figure 1: Reads per first copies.

For each of the 5 libraries, we show the reads per first copy distributions used to establish a cutoff for minimum reads for a first copy to be well-covered. Each first copy has a point on the graph, with the x-axis indicating its position in a sorted list by number of reads, and the y-axis indicating the number of reads assigned to that first copy.

Supplementary Figure 2: Bench protocol for partial mutagenesis and panel-based enrichment

In step 1, genomic DNA is fragmented, end-polished, and A-tailed. In step 2, the fork-tailed adapters are ligated. Step 3 involves hybridization capture with a set of biotinylated oligos that flank targeted microsatellite loci. In step 4, partial bisulfite treatment is applied, aiming for 50% conversion of C's to U's. Step 5 involves multiple rounds of linear amplification using oligo (1), which adds the linear copy varietal tag and sequencing adapter. Step 6 is one cycle of linear amplification to add the second sequencing adapter. Finally, step 7 proceeds with size selection followed by exponential PCR. The libraries are sequenced then sequenced on an Illumina sequencer (step 8).

Supplementary Figure 3: Sequencing base quality and base composition.

For each of the 5 libraries, we split the raw sequencing reads into 4 sets, based on read number 1 or 2, and base composition of the microsatellite (A vs. T, C vs. G, CA vs. TG). For each set of reads, we show the per base Phred-scale quality score, as well as the per base composition of A/C/G/T. In the base quality plot, the blue line indicates mean base quality; the red line shows median base quality; the box plot shows the 25th and 75th quantiles in yellow and the 10th and 90th quantiles in black. The microsatellite region is highlighted in blue in both plots. Base quality drops dramatically after reading through the mononucleotide microsatellites. This impact on base quality is not observed in the mutated libraries.

Supplementary Figure 4: Drop-out and matching tract lengths.

For each of the unmutated mononucleotide tracts, M-17 (A-) and M-18 (C-), we show a scatterplot of the measured lengths from the two reads in the pair, with the length measured from the read reading the mono tract as A or C track on the x-axis and the length measured from the read reading the mono tract as a T or G track on the y-axis. The -1 position is reserved for those cases where both strands were unable to make a microsatellite call. This plot contains a down-sampling to 1 million data points.

Supplementary Figure 5: Read coverage distribution over panel loci.

For the mutated panel library, we record the number of reads mapping to within 1000 bases of the microsatellite locus. These reads account for 72.4% of the library and are distributed with uniformity across all loci. Here we show the counts of reads restricted to autosomal loci for the C-panel and AC-panel separately.

Supplementary Table 1: Sequence information of the templates and the oligonucleotides

The sequences for the 3 template sequences: M-18 (C), D-26 (CA), and M-17 (A) are listed. N's indicate random nucleotides; D's indicate random nucleotides excluding C. Oligo (1) and primer sequences UP1 and UP3 are also listed for the two protocols used.

Supplementary Table 2: Disruption yields as a function of mutation rate

For the M-18 (C+) and D-26 (CA+) templates, the proportion of reads passing each of the two disruption parameter thresholds is modeled as a function of overall mutation rate. The reads that pass the rate cutoff are the reads with between 15% to 85% of C's mutated, and the reads that pass the repeat length cutoff have 5 or fewer units of the repeat intact. Highlighted in green are the mutation rate bins that most closely match the observed data.

Supplementary Table 3: Sequence information of the adapters and the oligonucleotides for panel enrichment protocol

The sequences for the 3 pairs of oligonucleotides to form 3 fork-tailed adapters are listed. D's indicate random nucleotides excluding C; /5Phos/ means 5' phosphorylation which is needed for DNA ligase. Panel-Oligo (1) and panel-oligo (2) are also listed. N's indicate random nucleotides.

Supplementary Table 4: Sequence information of the hybrid capture oligos for the C and AC panels

Oligos used for hybrid capture enrichment of microsatellite loci. Each locus has a 60 base 3 prime and a 60 base 5 prime oligo that flank the microsatellite sequence.

Supplementary Table 5: Companion table to Figure 3, per template first copy on- and off-target counts

The data underlying Figure 3, for the libraries M-18 (C++), D-26 (CA-), and D-26 (CA++), is shown. Each template is tabulated by the number of well-covered first copies on-target (equal to 18 or 26) and the number of well-covered first copies off-target. For the disrupted libraries, templates have either all first copies on-target or all first copies off-target. For the D-26 (CA-), many templates are mixed with both on-target and off-target first copies.

Supplementary Table 6: Lengths of unanimous templates

For the three libraries, M-18 (C++), D-26 (CA-), and D-26 (CA++), shown in Figure 3, the number of templates with unanimous first copies, for each possible length, is tabulated, separated further by the number of first copies per template. The vast majority of unanimous templates are of the expected on-target length (18 or 26). Synthetic variant templates (3+ unanimous first copies) were removed before the final error rate estimations.

Supplementary Table 7: Error rates as a function of disruption parameters

For the two mutated libraries, we analyze the read off-target rate, and number of reads retained, as a function of the two disruption parameters. The max residual repeat length is varied from 3 to 18 for the M-18 library and 3 to 13 for the D-26 library, with the conversion rate thresholds varying from 0.45 to 0.55 as the strictest threshold, to 0.05 to 0.95 to the loosest threshold. Highlighted in green are the values for the parameter range in our main analysis. Error rates can be reduced further by using stricter disruption thresholds, though yield is also affected by this choice.

Supplementary Table 8: k-multiplet unanimity rates for synthetic variant templates and cell-line panel data

Following the format of Table 2, in table (A) we perform the same calculations restricted to those templates previously determined to be synthetic variants. For table (B) we perform the same calculations aggregating over all disrupted templates in the panel data, but restricting to those within a similar size range to the synthetic templates in Table 2. CA++ panel results are limited to templates with length 21 to 31. C++ panel results are limited to templates with length 13 to 23.

Supplementary Table 9: MSL counts for all panel loci

Counts of triplet observations for all panel loci including those in Figure 6, for both unmutated and disrupted libraries. Microsatellite length of "M" indicates a mismatch among reads in the triplet. Positions are relative to the hg38 reference genome.

Supplementary Figure 1

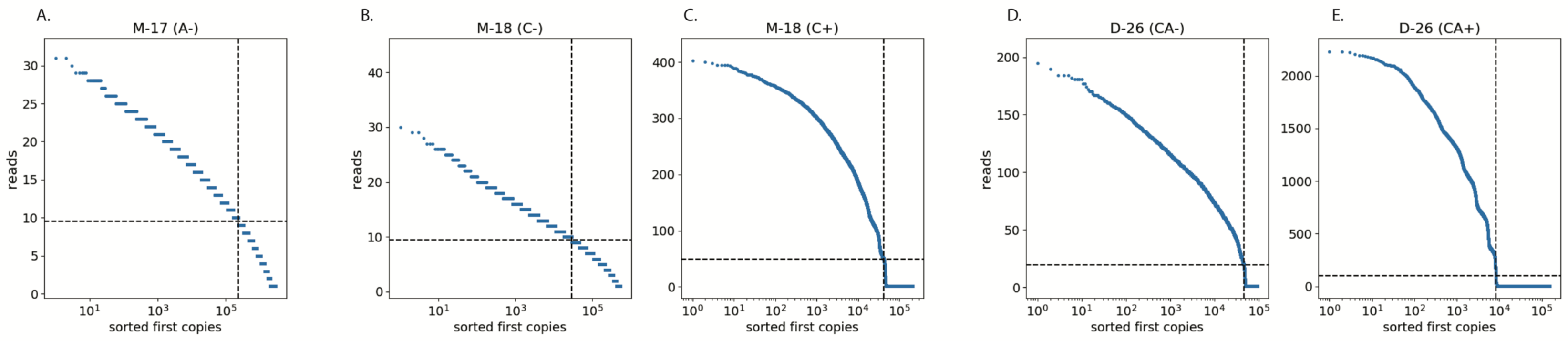

Supplementary Figure 1: Reads per first copies. For each of the 5 libraries, we show the reads per first copy distributions used to establish a cutoff for minimum reads for a first copy to be well-covered. Each first copy has a point on the graph, with the x-axis indicating its position in a sorted list by number of reads, and the y-axis indicating the number of reads assigned to that first copy.

Supplementary Figure 2

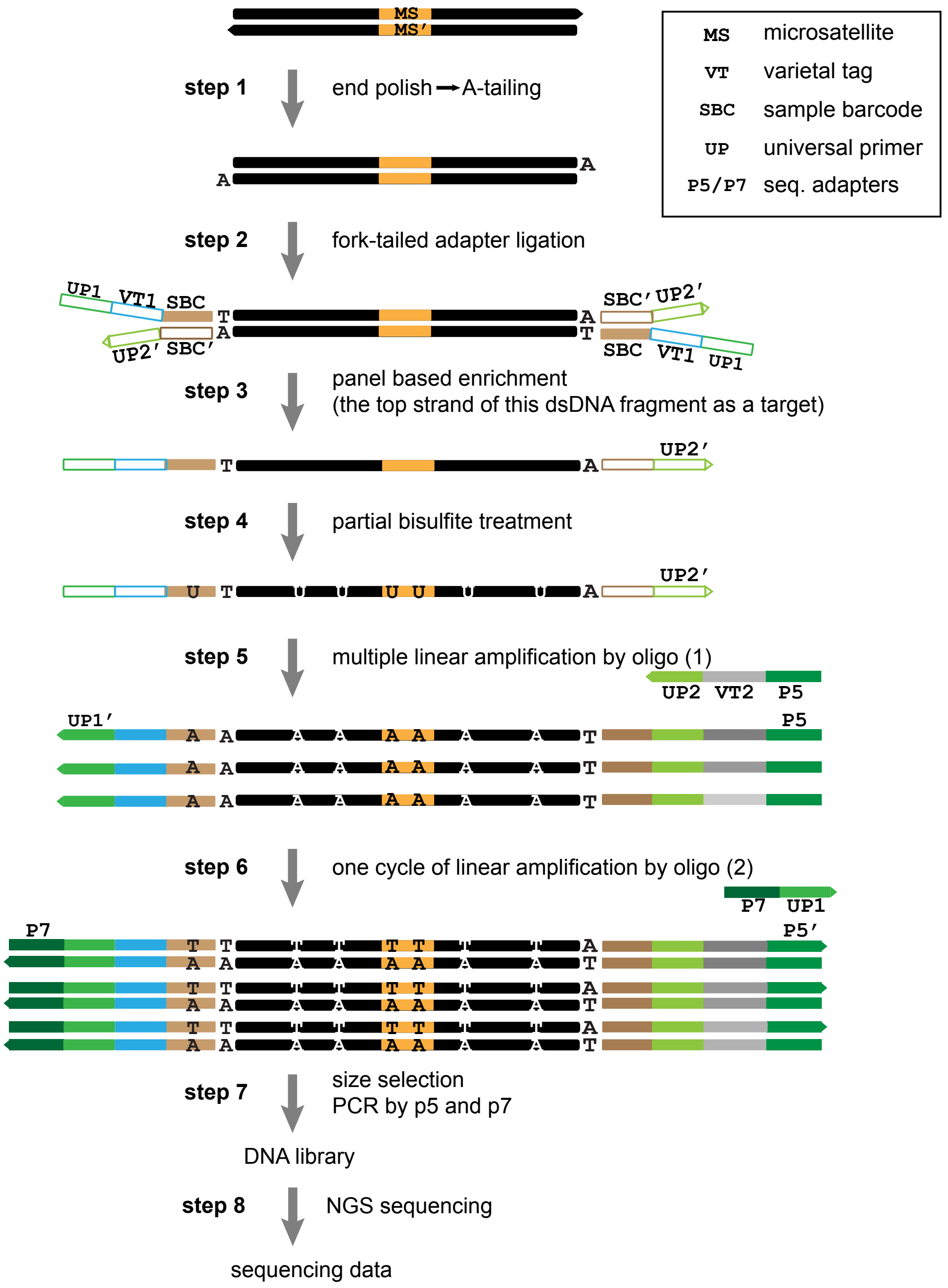

Supplementary Figure 2: Bench protocol for partial mutagenesis and panel-based enrichment. In step 1, genomic DNA is fragmented, end-polished, and A-tailed. In step 2, the fork-tailed adapters are ligated. Step 3 involves hybridization capture with a set of biotinylated oligos that flank targeted microsatellite loci. In step 4, partial bisulfite treatment is applied, aiming for 50% conversion of C's to U's. Step 5 involves multiple rounds of linear amplification using oligo (1), which adds the linear copy varietal tag and sequencing adapter. Step 6 is one cycle of linear amplification to add the second sequencing adapter. Finally, step 7 proceeds with size selection followed by exponential PCR. The libraries are sequenced then sequenced on an Illumina sequencer (step 8).

Supplementary Figure 3

Sequencing Base Quality and Base Composition

Annotated Example:

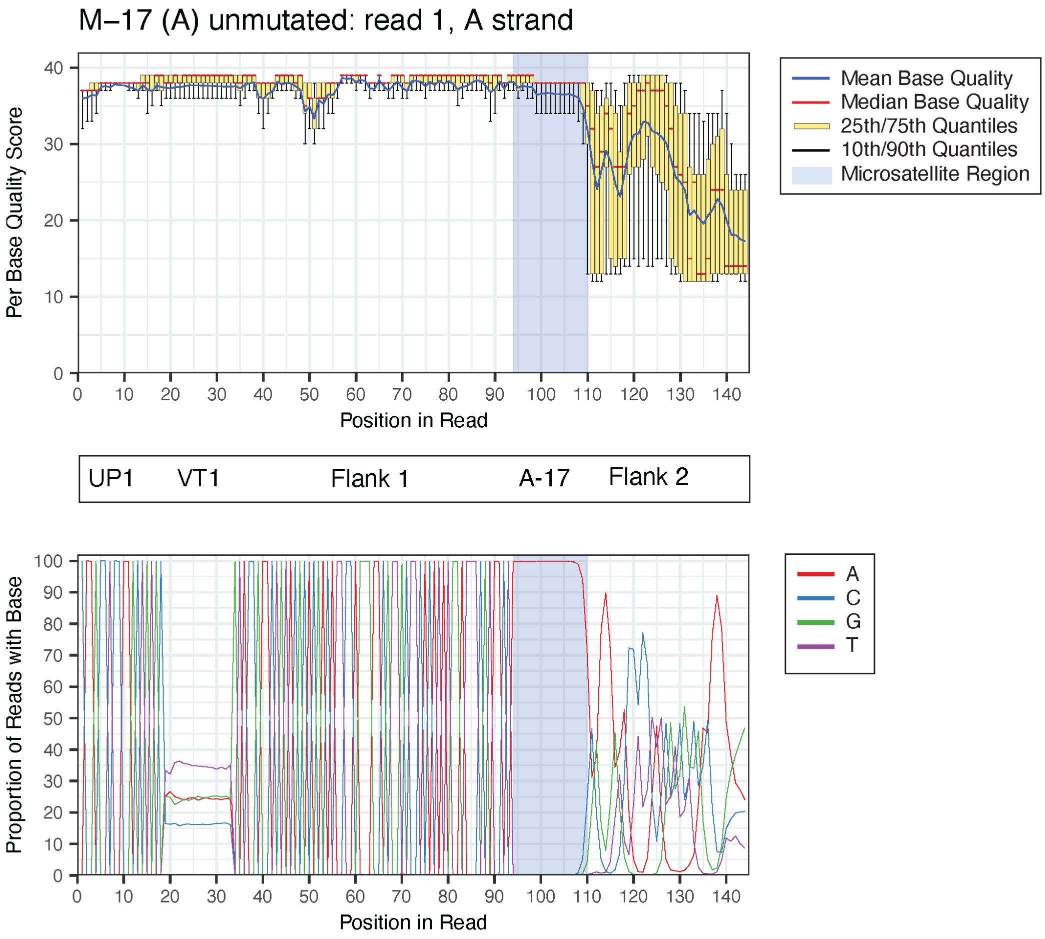

Supplementary Figure 3: Sequencing base quality and base composition. For each of the 5 libraries, we split the raw sequencing reads into 4 sets, based on read number 1 or 2, and base composition of the microsatellite (A vs. T, C vs. G, CA vs. TG). For each set of reads, we show the per base Phred-scale quality score, as well as the per base composition of A/C/G/T. In the base quality plot, the blue line indicates mean base quality; the red line shows median base quality; the box plot shows the 25th and 75th quantiles in yellow and the 10th and 90th quantiles in black. The microsatellite region is highlighted in blue in both plots. Base quality drops dramatically after reading through the mononucleotide microsatellites. This impact on base quality is not observed in the mutated libraries.

A.

M-17 (A-) unmutated

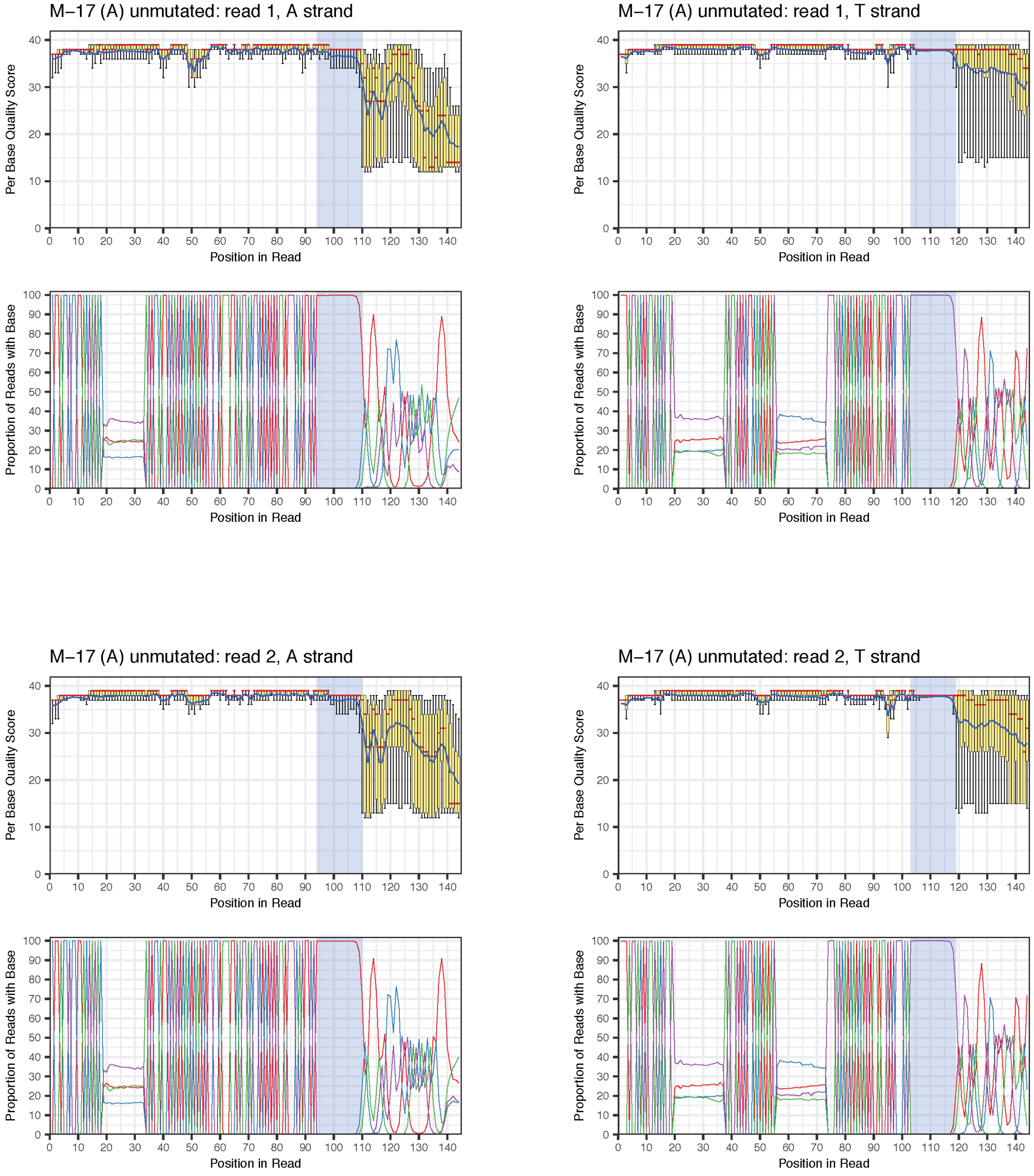

B.

M-18 (C-) unmutated

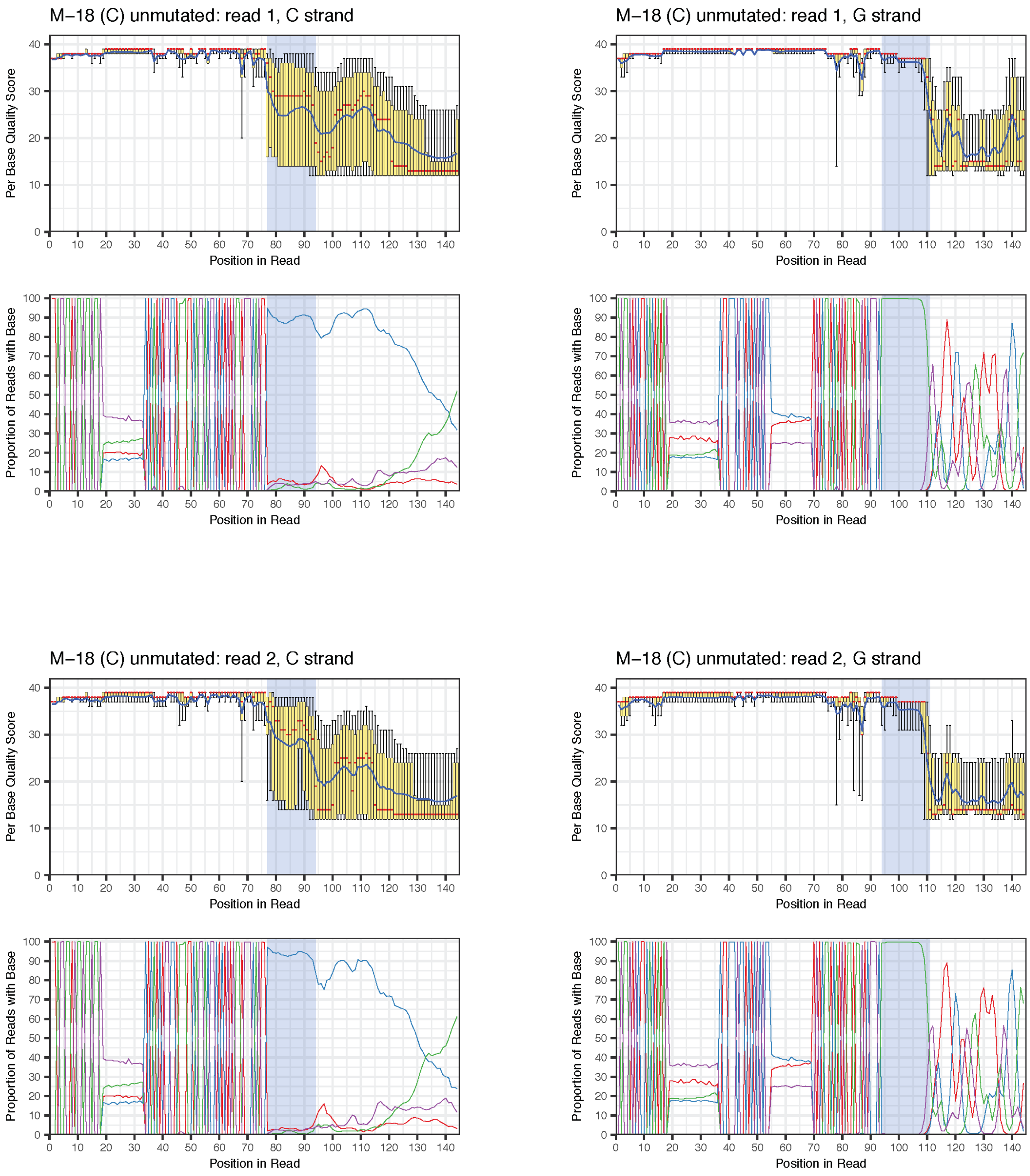

C.

D–26 (CA-) unmutated

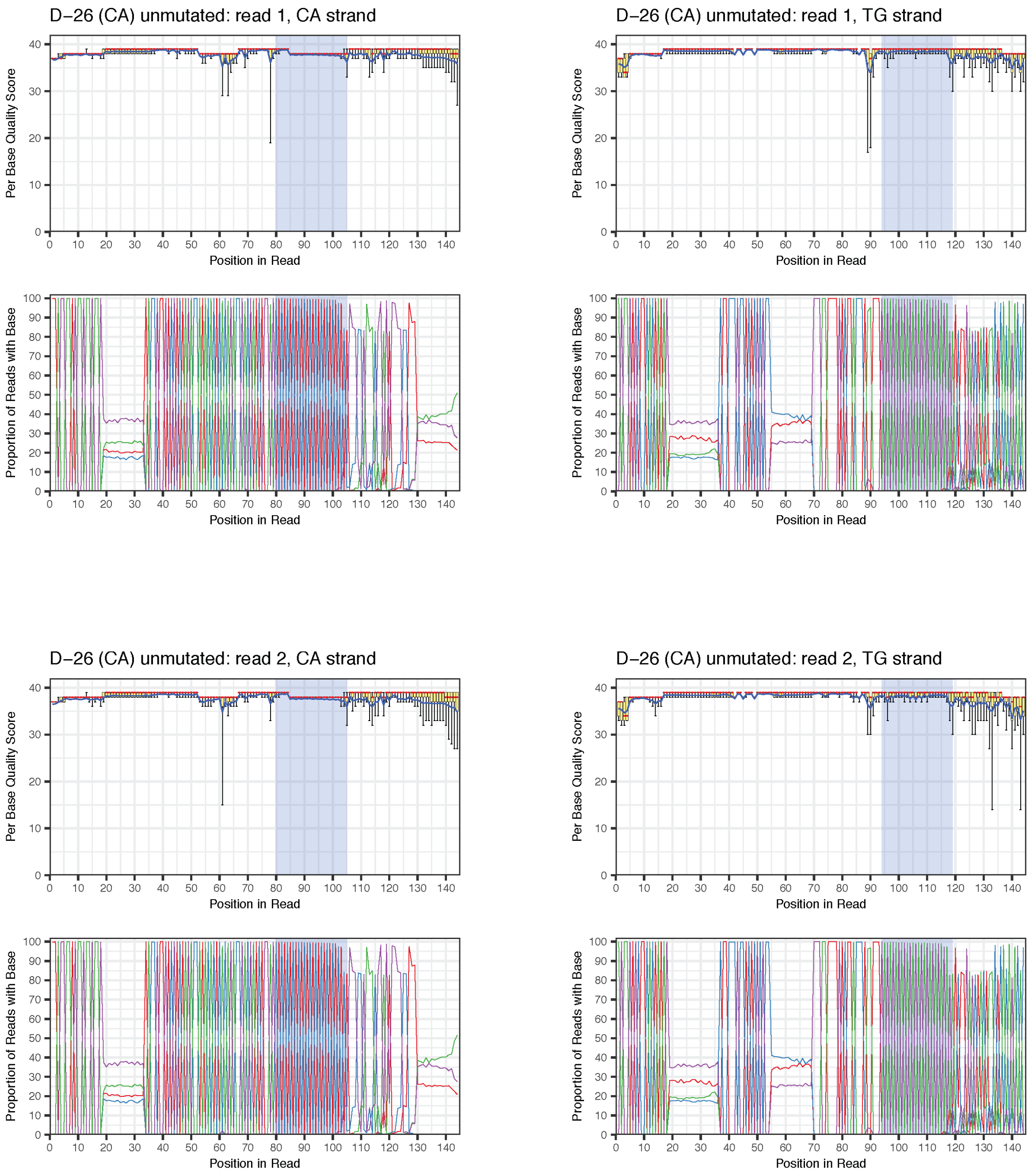

D.

M-18 (C+) mutated

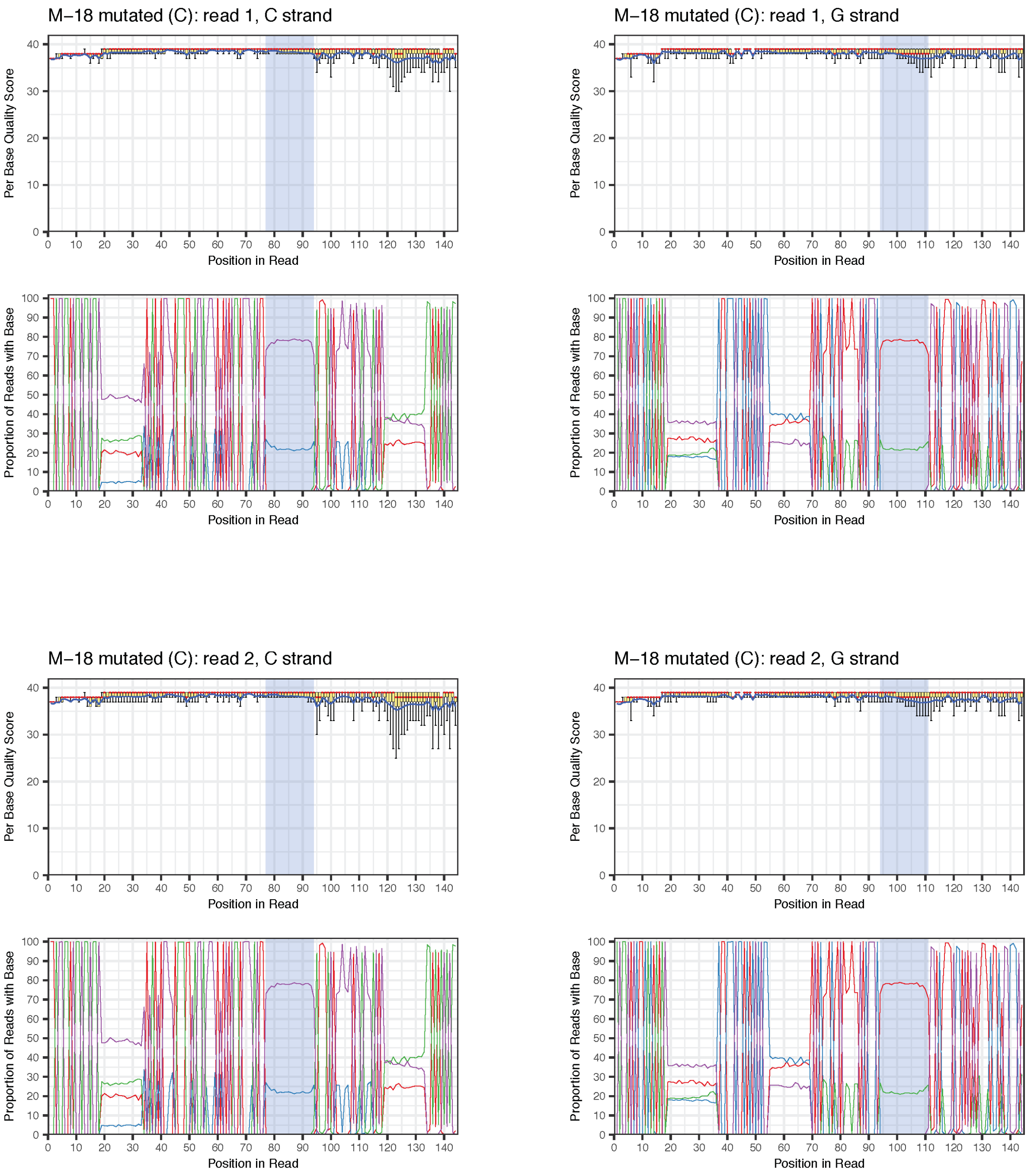

E.

D–26 (CA+) mutated

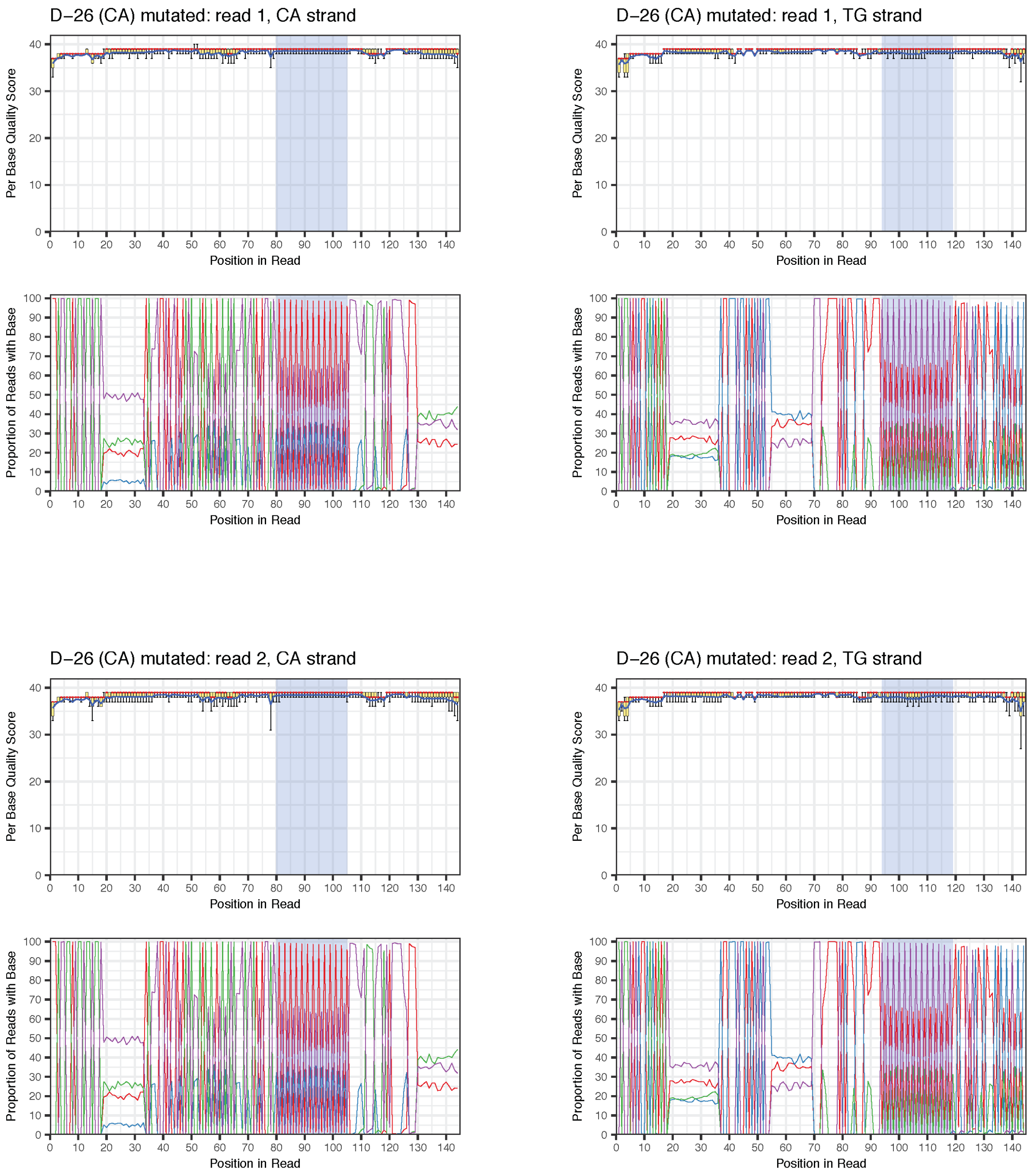

Supplementary Figure 4

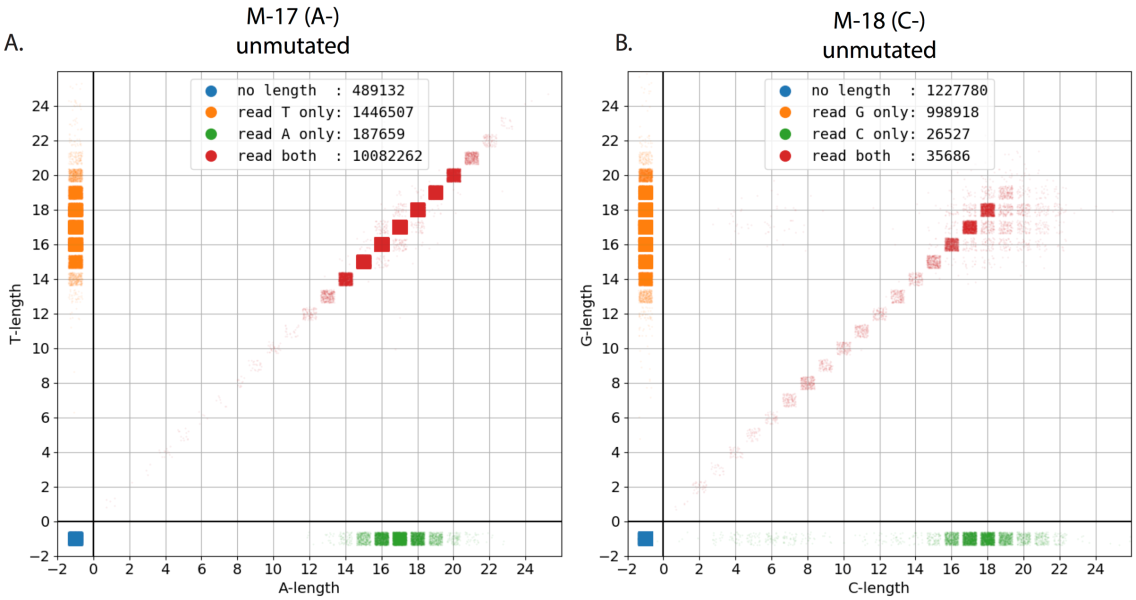

Supplementary Figure 4: Drop-out and matching tract lengths. For each of the unmutated mononucleotide tracts, M-17 (A-) and M-18 (C-), we show a scatterplot of the measured lengths from the two reads in the pair, with the length measured from the read reading the mono tract as A or C track on the x-axis and the length measured from the read reading the mono tract as a T or G track on the y-axis. The -1 position is reserved for those cases where both strands were unable to make a microsatellite call. This plot contains a down-sampling to 1 million data points.

Supplementary Figure 5

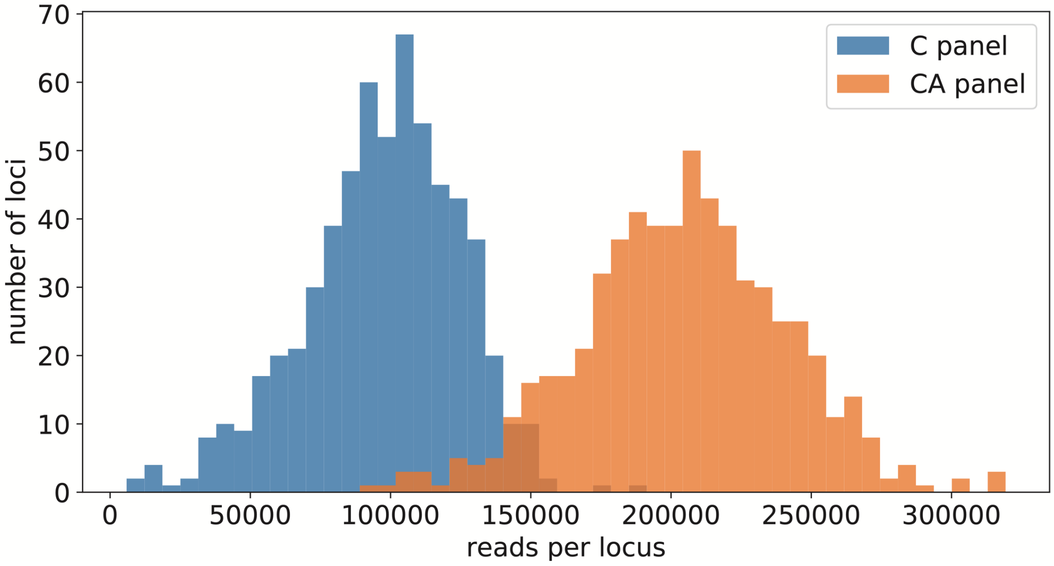

Supplementary Figure 5: Read coverage distribution over panel loci.

For the mutated panel library, we record the number of reads mapping to within 1000 bases of the microsatellite locus. These reads account for 72.4% of the library and are distributed with uniformity across all loci. Here we show the counts of reads restricted to autosomal loci for the C-panel and AC-panel separately.
